# Density Contrast Sedimentation Velocity for the Determination of Protein Partial-Specific Volumes

**DOI:** 10.1371/journal.pone.0026221

**Published:** 2011-10-20

**Authors:** Patrick H. Brown, Andrea Balbo, Huaying Zhao, Christine Ebel, Peter Schuck

**Affiliations:** 1 Biomedical Engineering and Physical Sciences Shared Resource, National Institute of Biomedical Imaging and Bioengineering, National Institutes of Health, Bethesda, Maryland, United States of America; 2 Dynamics of Macromolecular Assembly Section, Laboratory of Cellular Imaging and Macromolecular Biophysics, National Institute of Biomedical Imaging and Bioengineering, National Institutes of Health, Bethesda, Maryland, United States of America; 3 Institut de Biologie Structurale, Université Grenoble 1, Grenoble, France; 4 Centre National de la Recherche Scientifique, Grenoble, France; 5 Commisariat à l'Energie Atomique, Grenoble, France; University of South Florida College of Medicine, United States of America

## Abstract

The partial-specific volume of proteins is an important thermodynamic parameter required for the interpretation of data in several biophysical disciplines. Building on recent advances in the use of density variation sedimentation velocity analytical ultracentrifugation for the determination of macromolecular partial-specific volumes, we have explored a direct global modeling approach describing the sedimentation boundaries in different solvents with a joint differential sedimentation coefficient distribution. This takes full advantage of the influence of different macromolecular buoyancy on both the spread and the velocity of the sedimentation boundary. It should lend itself well to the study of interacting macromolecules and/or heterogeneous samples in microgram quantities. Model applications to three protein samples studied in either H_2_O, or isotopically enriched H_2_^18^O mixtures, indicate that partial-specific volumes can be determined with a statistical precision of better than 0.5%, provided signal/noise ratios of 50–100 can be achieved in the measurement of the macromolecular sedimentation velocity profiles. The approach is implemented in the global modeling software SEDPHAT.

## Introduction

The protein partial-specific volume 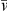 is a key parameter in the interpretation of data from biophysical techniques such as analytical ultracentrifugation, dynamic light scattering, and small-angle scattering [1]–[3]. Basically, sedimentation equilibrium analytical ultracentrifugation (SE) and sedimentation velocity analytical ultracentrifugation (SV) require the particle partial-specific volume to predict its buoyancy (i.e., density increment ∂*ρ*/∂*c*) and to obtain absolute molar mass values and molar mass distributions [4]. Although some types of studies of macromolecular interactions can be carried out without prior knowledge of the partial-specific volumes, such as isotherms of weighted-average sedimentation coefficients [5], [6] and isotherms of reaction boundary s-values [7], [8], it is very important, for example, in the analysis of the oligomeric state of proteins and protein complexes, including detergent-solubilized membrane proteins, and their self-associations. Further, the value of the partial- specific volume indeed reflects particle composition, and flexible methods for the determination of partial-specific volumes are required in the characterization of novel biomaterials, nanoparticles, and covalent protein/polymer conjugates in biotechnology [9]–[13]. There is a requirement for high precision of the partial-specific volume data, since the error on the partial- specific volume typically propagates as an error of three times more in the molar mass as determined from sedimentation, for a soluble protein in dilute aqueous buffer, and much more for less dense macromolecules or assemblies.

Also, hydrodynamic methods require knowledge of the protein volume in order to determine hydrodynamic frictional ratios that relate measured quantities to shape. The importance of methods for determining the partial-specific volume of macromolecules in this area has increased with the availability, on one hand, of more precise techniques for the prediction of protein frictional coefficients from available structural data [14], and on the other hand, the increased precision in the determination of sedimentation coefficients in analytical ultracentrifugation accompanied by modern direct-boundary modeling techniques [15].

While the average partial-specific volume of all known human protein sequences has a value of 0.735 ml/g [16], there is a significant variation dependent on amino acid composition. The apparent partial-specific volume of dissolved proteins is further modulated by covalent protein modifications, such as glycosylation, and also reversible ligand binding and solvation, thus carrying significant information, besides particle composition, about interactions with solvent and co-solutes [17]–[19]. Various methods for its experimental determination or theoretical estimation have been developed, including [20]–[33].

Since the amino acid sequences of proteins are usually available, the most convenient approach for estimating partial-specific volumes 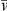 is the compositional prediction [20], [21]. However, this is not sufficient for calculating the density increment ∂*ρ*/∂*c*, when non-covalent interactions become important. This is the case, for example, when proteins are studied in the presence of multi-component solvents where the hydration shell is not of equal composition as the solvent [18]. This should be expected with highly charged macromolecules [34] that are associated with a significant number of counter-ions, with proteins binding small ligands such as denaturants or detergents [35], or, *vice versa*, for proteins under conditions where the hydration shell specifically excludes some solvent components [19], [36]. In these cases, it is useful to make the operational distinction whether or not the mass and/or volume of the solvation shell should be counted towards the suspended sedimenting particle, which consequently leads to definitions of different partial-specific volumes (see below). Hydration can significantly contribute to the density increment in analytical ultracentrifugation, and produce large changes in the effective buoyancy of proteins, when studied in buffers that have densities far from that of water. On the other hand, sedimentation in the presence of a densifying co-solvent that is excluded from the hydration shell can provide a tool to probe the degree of protein hydration *via* measurement of the buoyant molar mass in SE [36]. While the extent of hydration/co-solvent binding is not always known *a priori* to permit a predictive approach, data and strategies for estimating contributions from some co-solutes are available [36]–[38].

Another convenient approach is the experimental ultracentrifugal determination directly of the density increment ∂*ρ*/∂*c* in SE or SV exploiting a known exact molar mass from sequence or mass spectrometry. Unfortunately, the experimental determination can fail in samples that do not have sufficient purity, an unknown oligomeric state, or exhibit reversible self-association in the accessible concentration range. Further, (similar to the compositional prediction) this approach is intimately connected with our definition of what fruitfully can be considered the sedimenting particle. Especially in the presence of non-covalent interactions with ligands or co-solvents, translating density increments ∂*ρ*/∂*c* into 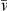 values is not directly possible (see below).

A method that can circumvent many of these difficulties, arguably the gold standard, is the macroscopic measurement of the density increment with a Kratky balance [26], [28], [39]. It has the virtue that it is straightforward to be carried out strictly following the thermodynamic prescription of dialysis equilibrium of the protein with the solvent [40]–[42]. Unfortunately, a major drawback is that for accurate measurements between 5–20 mg of pure protein are required at concentrations up to several mg/ml [29], [40], [43]. Very often in contemporary studies, such quantities and/or concentrations of fully dissolved, non-aggregated protein are experimentally out of reach.

An elegant strategy for determining the apparent partial-specific volume and simultaneously the molar mass of a protein with microgram quantities of material at moderate concentration has been demonstrated by Edelstein and Schachman [25]. It is based on the idea of exploiting a density contrast, by measuring the buoyant molar mass of a protein in SE in both H_2_O and D_2_O buffers. Reynolds & Tanford have pioneered a related density matching approach for studying detergent solubilized membrane proteins [35], and further extensions to detergent solubilized membrane proteins with prosthetic groups have been reported [44]. Density variation SE has also been applied to G-quadruplex DNAs [45]. In modern variations, it has been implemented for direct global non-linear regression [46], [47] (though not yet accounting for corrections due to H-D exchange, see below).

Unfortunately, a significant limitation of density contrast SE is its requirement for highly pure and non-associating material, since for heterogeneous samples the measured cell-average buoyant molar mass would be skewed to higher numbers in higher density solvents, an error that can be greatly amplified due to the usually long extrapolation from the range of experimental solvent densities to the density of the protein. Variations of this density contrast SE approach can lead to a larger solvent density range, using buffers with the very expensive D_2_O^18^, or using densifying co-solvents [48]–[50], the latter with the concomitant potential concern of encountering preferential solvation effects that can limit the accuracy [33], [42], [43]. Recently, it has been demonstrated by Rowe and colleagues [51] that H_2_O^18^ , which has become commercially available at non-prohibitive cost, can simplify density contrast experiments by providing the same density contrast as D_2_O, without the complications of accounting for H-D exchange altering the molar mass of the particles of interest in the different buffers.

The principle of density contrast approach has also been described for sedimentation velocity analytical ultracentrifugation (SV) [11], [22]–[24], [31], [33], [35], [52] [53], [54]. While only requiring slightly more protein than SE, this method has the potential to be suitable for polydisperse systems, and the virtue of not as stringent sample purity requirements. Recently, Gohon *et al.* have shown how the high resolution and accuracy of species' *s*-values from diffusion-deconvoluted sedimentation coefficient distributions *c(s)* can be exploited to determine the partial-specific volume of amphipols with high precision [33]. Inspired by this work, we aimed at simplifying and further improving the precision of density contrast SV for the apparent partial-specific volume through implementing the global non-linear regression sedimentation velocity data measured in different solvents, with the protein partial-specific volume as an adjustable parameter jointly with the determination of the protein sedimentation coefficient distribution. In principle, by directly fitting the entire sedimentation boundaries including the diffusional spread, information from both the differential buoyant molar mass and the differential sedimentation velocity can be exploited. We present data from density contrast SV of three different proteins in H_2_O/H_2_^18^O mixtures, and critically compare the best-fit estimates with values determined from other methods.

## Materials and Methods

The theoretical background is well-known but recapitulated here for context. Necessarily, in this brevity it remains superficial, and for more details see [3], [18], [41], [55] and others.

### Partial-specific volume

The partial-specific volume (ml/g) is a measure of the inverse of the density of the species. It is rigorously thermodynamically defined from the increase of the volume, *V* (ml),of the solution when adding a small amount (*w*, in gram) of species, at constant pressure and temperature, the amounts (*w*_i_) of all the other components present in the solution being held constant.
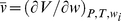
(1)The partial-specific volume is determined mainly by the effective volume occupied by the species, while its value depends also on volume changes induced by the compound on the other components in the solution. For example, the partial-specific volume of salts or nucleic acids increases when solvent salt concentration in increased, because electrostriction of water is less pronounced at high salt (see e.g. [56]). For proteins, the combination of partial-specific volume estimates from experimental density measurements on solubilized individual amino acids [20] allows reliable estimates of partial specific volumes of proteins. The summation of the volumes of packed buried amino acids as determined in protein crystal leads, when adding negative contributions for water electrostriction of 10 and 18 Å^3^ by acidic and basic residues, to the experimental values of partial-specific volumes of proteins, because all other volume changes upon protein folding compensate [57]. The value of the partial-specific volume is directly related to the definition of the particle, i.e. what is considered in *w*, in the equation above, which is usually anhydrous macromolecule or macromolecular complex.

### Buoyancy, density increment, and partial-specific volume

The sedimentation of two-component (macromolecule and solvent) systems in the limit of low macromolecular concentration is governed by the Svedberg relationship
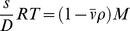
(2a)
[58] which relates the sedimentation coefficient *s*, the diffusion coefficient *D*, the molar mass *M*, and the macromolecular partial-specific volume 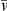. The right-hand-side of Eq. 2a is also termed buoyant molar mass. The buoyancy term 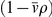 is the density increment 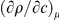 (with concentration *c* at constant chemical potential μ), which may be measured experimentally:
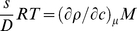
(2b)In the presence of multi-component solvents and for charged macromolecules, Eq. 2a is invalid and Eq. 2b should be used. The density increment at constant chemical potential can be written using preferential binding parameters, or, alternatively, in the description of a particle [59]:
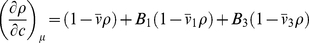
(3)which accounts for the binding of solvent (subscript 1) and co-solvent (subscript 3) to the macromolecule (usually subscript 2, here noted without subscript) [41]. *B* are the binding parameters (in g/g), which in the invariant particle model (i.e., if the particle has the same composition in solvents of different composition [59]), are macromolecular constants.

The measured buoyant molar mass is then
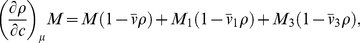
(4)i.e. composed of contributions of *M_1_ = MB_2_* solvent and *M_3_ = MB_3_* co-solvent (gram per mol of macromolecule), in addition to that of the macromolecule. Therefore, it is straightforward and physically intuitive to define the sedimenting particle (index *sp*) (or ‘equivalent particle’ [1]) as being the composite of all linked pieces, though non-covalently linked, which would then have the total molar mass
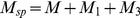
(5)and the weight-average partial-specific volume 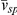


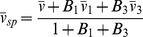
(6)which is invariant only if the invariant particle model holds [1]. More generally, it could comprise at least the entire volume from where the solvent is disturbed from that in the bulk [2], [3]. In this way, the simple form of the Svedberg equation is re-established as
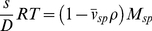
(7)Considering the invariant particle model conceptually greatly facilitates the incorporation of spectral and hydrodynamic information [60]–[62]. The Svedberg equation in the form of Eq. 7 is usually applied for “strong” complexes, such as protein/protein, protein/nucleic acid, or even protein/detergent complexes in diluted solvents [62]–[64]. Note, however, that for membrane proteins, when lipids are contributing to the complex in undefined amounts, Eq. 2b can be used more advantageously, since 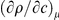 can be measured.

Particle definition is a matter of choice, usually decided according to the experimental setup. It would be unusual (though correct) for a 100 kg/mol protein in dilute solvent that is hydrated with 0.3 g/g - as probed in a more complex solvent - to be thought of as a sedimenting particle of having a molar mass of 130 kg/mol and a 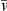 of 0.793 ml/g (assuming a polypeptide 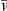 of 0.730 ml/g). The buoyant molar mass would be the same considering a much larger value for the hydration. Clearly, as long as the preferentially bound solvent component is close to neutrally buoyant, such as hydration in buffers of density not far from water, 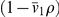 is very small and therefore 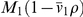 can be negligible despite a large *M_1_*.

In general, the effect of hydration on the buoyant term can be evaluated in Eq. 3. Formally, we may also account for the deviations between 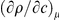 and 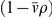 by introducing an ‘apparent’ partial-specific volume 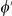:
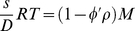
(8)with
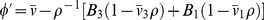
(9)The apparent partial-specific volume 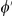 is an operationally defined quantity and not a molecular constant, and is dependent on the preferential binding parameters and the buffer density. It will be identical to the macromolecular 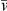 only if no co-solute is bound or if the bound co-solute is neutrally buoyant. For example, the same neutral and hydrated 100 kg/mol protein with 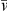 of 0.730 ml/g would appear to have a 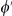-value of 0.733 ml/g in a solvent density of 1.01 g/ml, but a 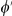-value of 0.757 ml/g in a solvent density of 1.10 g/ml. The latter corresponds to a relatively large amount of co-solvent, such as either 2.3 M KCl, 2.5 M NaCl, 1.4 M (NH_4_)_2_SO_4_, 25% sucrose, or 40% glycerol. Potentially, if hydration is neglected, i.e. 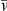 used instead of 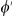, this would lead to errors in the determination of *M* of 1.1% in the 1.01 g/ml buffer and 15.2% in the 1.10 g/ml buffer, respectively. For this reason, the approximation of 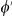 by a compositional 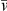 is common practice and usually a reasonable approximation for proteins of little charge in dilute buffers [25], [43], but would lead to qualitative errors for highly charged macromolecules and/or proteins in buffers containing densifying co-solutes such as sucrose or glycerol that could potentially be unacceptable, dependent on application. The hydration terms may be particularly important for detergent-protein complexes where 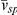 is closer to 1.0 ml/g.

(For completeness, we want to mention that we often use for the study by analytical ultracentrifugation of heterogeneous interactions between macromolecules without volume change in complex formation, an arbitrarily defined 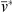 , in order to consistently scale the measured buoyant molar masses *M_b_* to more familiar molar mass 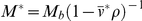.)

It is important to note that different methods determine different quantities: While from densimetry of proteins in dialysis equilibrium we obtain 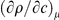 where *c* refers to the anhydrous protein concentration; compositional prediction leads to 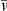; and the buoyant molar mass using a known protein molar mass provides 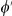. Further, from density contrast experiments of proteins using for the modulation of solvent density a co-solute that is excluded from the hydration shell (as generally considered in the presence of sucrose, glycerol, sodium, or potassium chloride) we will get 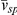 (i.e. the full mass and partial-specific volume of the hydrated particle as distinguished from the solvent), which can also be expressed in terms of *M*, 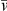, and *B*_1_ (restricting to the systems with *B*_3_ = 0). On the other hand, the modulation of solvent density can be achieved in a way that maintains neutral buoyancy of the hydration shell, when using dilute buffers enriched in water with heavy hydrogen and/or oxygen isotopes. Then, sedimentation is achieved in a two-component system (water and macromolecule), which provides directly *M* and 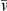 of the macromolecule [25] (again assuming negligible *B_3_* [25], the opposite being relevant only for polyelectrolytes [33] [65] [56], [66] or for systems comprising ligand or detergent binding to the macromolecule, which will not be considered in the present work).

### H-D exchange

Complications arise when using water containing deuterium for density contrast due to the H-D exchange of exchangeable hydrogens in the protein by deuterium [25], [33], [35], [63]. With *k* customarily denoting the molar mass ratio in deuterated relative to that in non-deuterated solvent, the measured buoyant molar mass in D_2_O solutions can be written as
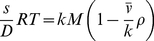
(10)(Here, *M* and 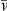 are given per gram of hydrogenated macromolecule, and are thus invariant with the solvent composition.) An average value of *k* of 1.0155 in 100% D_2_O is given in [24], [25], thought to be relatively constant for all proteins. In solvents containing only a percentage of D_2_O, the value of *k* is proportionally reduced. This will be expressed as 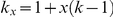 where *k_x_* is the ratio of protein molar mass in the partially deuterated buffer relative to non-deuterated buffer, and *x* in this context is the molar fraction of D_2_O of all the water in the buffer. Clearly, H-D exchange can be avoided by using H_2_^18^O rather than D_2_O as solvent [51], the two solvents providing similar solvent densities (*k* = 1.0 in H_2_O or H_2_^18^O).

### Analytical description of density variation analysis

The concept of density variation analysis is first presented in its analytical form [33], [45]. Although this is computationally different from the route explored in the present work, it presents the information flow of density variation analysis more transparently and allows for a straightforward error analysis.

We assume two AUC experiments, one in low density solvent (H_2_O), and one in high density (such as D_2_O). As described by Gohon *et al.* [33], the effect of density variation on the macromolecular sedimentation can be captured best in the quantity *R*, defined as
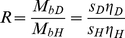
(11)(with *η* the solvent viscosity, and the subscript ‘D’ and ‘H’ indicating H2O and D2O solvent, respectively). If co-solvent interactions are unchanged in both solvents, we can obtain the partial-specific volume as
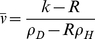
(12)If the error 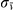 in determining 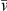 that way were to arise solely from errors 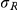 in R, we would have
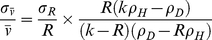
(13)For proteins with 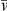 = 0.73 ml/g in solvents without H-D exchange of density 1.006 and 1.103 g/ml (compare Table 1), *R* is 0.7334, and relative errors in the determination of *R* will be amplified with the factor 0.7308. *Vice versa*, in order to determine 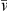 with a precision of 1%, *R* would need to be determined with a precision of ∼1.4% or better. This level of precision in relative *s-*values can be experimentally achieved easily, and may be surpassed in careful experimentation by a factor 10 [67]. On the other hand, for example, metal nanoparticles with 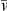 = 0.15 ml/g would have a *R*-value of 0.9806, and a 1% error in *R* would result in 42% error in 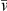.

**Table 1:** Density and viscosity values measured for PBS solutions containing different fractions of H_2_^18^O.

H_2_^18^O fraction(a)	0	0.5	0.9	0	0.5	0.9
temperature (°C)	density (g/ml)			viscosity (P)		
20	1.005584	1.059388	1.102733	0.010219	0.010456	0.010665
16	1.006406	1.060246	1.103606	0.012230	0.011493	0.011725
10	1.007304	1.061186	1.104581	0.013136	0.013436	0.013716
4	1.007758	1.061652	1.105048			

(a) H_2_^18^O fraction by volume, using the 97% isotopically enriched heavy-oxygen water as a reference.

### Frictional coefficient and frictional ratio

The frictional ratio is the ratio of the experimentally determined translational frictional coefficient, *f*, of the hydrated, anisotropic macromolecule to the calculated frictional coefficient, *f*_0_, of a non-hydrated, smooth spherical macromolecule of the same molecular mass. *f*_0_ is calculated from 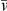 and M, considering the anhydrous volume is 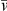 M/N_A_:
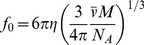
(14a)Thus, the frictional ratio has components from shape asymmetry and from hydration. Alternatively, because a multi-component particle can be arbitrarily defined, in the sedimenting particle picture of Eqs. 5 and 6, if the reference particle is a compact smooth hydrated sphere of molar mass and partial-specific volume of *M_sp_* and 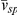, respectively,
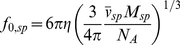
(14b)then the resulting frictional ratio *f/f_0, sp_* will have only shape contributions.

### Direct global density variation SV analysis with integral equations and sedimentation coefficient distributions

We assume that no change takes place in the association state and in the partial-specific volume, except for the effect of H-D exchange. Similarly, we assume a constant translational frictional ratio *f/f_0_* under different buffer conditions, and that all experiments are conducted at the same temperature. The goal is to express the sedimentation data globally as a distribution of *s*-values at standard conditions of water at 20°C (which is denoted in the following with the superscript 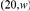, to avoid confusion with the numerical indices introduced below). When using the *c(s)* sedimentation coefficient distribution [68], a constant frictional coefficient *f/f_0_* is used to approximately scale the diffusion coefficient as a function of the sedimentation coefficient:
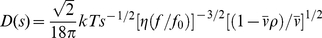
(15)
[68]. This equation is applied at standard conditions, to relate *D* ^(20,w)^, *s* ^(20,w)^, and *f/f_0_* ^(20,w)^. Since the sedimentation and diffusion coefficients are
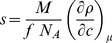
(16)and
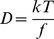
(17)a given s^(20,w)^-value translates to *s*-values at experimental conditions *s^(xp)^* as
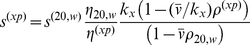
(18)
[23] (which is the well-known correction formula [4] if *k_x_* = 1 in non-deuterated buffer), and the diffusion coefficient translates as
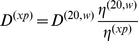
(19)It should be noted that when the 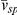 of the hydrated particle is probed with density contrast by densifying co-solutes that don't penetrate into the hydration shell, the frictional ratio as obtained here will likewise include hydration contributions (i.e. be 1.0 for an ideal compact hydrated protein, see above).

The computational analysis of the global *c*(*s*) method starts with a grid of *s^(20,w)^-*values, *s_i_* (*i* = 1, …, *I*), each corresponding to a putative species, chosen across a sufficiently wide range and with sufficient density so as not to constrain the quality of fit (for example, 100–150 values between 0 and 10 S for the study of BSA) [69], [70]. It also requires an initial guess of the average frictional ratio, which is to be adjusted in the global non-linear regression. It is used to calculate for each species the diffusion coefficients corresponding to each of the *s_i_*-value *via* Eq. 13. Transformation to the experimental condition of the experimental data set *n* (with a total number of SV experiments *N*) can then take place with Eqs. 16 and 17. Knowing the sedimentation coefficient 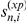 and diffusion coefficient 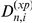 for the experimental conditions to be modeled, we can calculate each species' concentration evolution 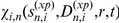 as a function of time *t* and radius *r* with the Lamm equation
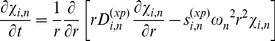
(18)
[71], where 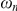 is the rotor speed at experiment *n*, with uniform loading concentration initially between meniscus *m_n_* and bottom *b_n_*. It can be solved best with the finite element method with obligate error control [72], in order to ensure that the numerical precision exceeds the experimental one, and to avoid excessive discretization errors [70]. These solutions form the kernel for the Fredholm integral equation relating the sedimentation coefficient distribution *c*(*s*),
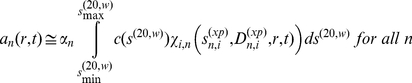
(19)to the experimental radius- and time-dependent signal *a_n_(r,t)* measured in data set *n*, which is rephrased as a discrete global least-squares model to all experimental data sets, after adding suitable baseline terms to account for systematic noise [73], [74]. Eq. 19 incorporates the scaling factors 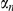 to accommodate possible small differences in concentration or specific signal increment in the different experiments, but constrains the sedimentation coefficient distribution to be unchanged. Similar to the previously described distribution models [68], [75], this leads to matrix equations for *c*(*s_i_*) and 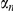. It is combined with Tikhonov-Phillips regularization to eliminate unreliable spikes that are not warranted by the information content of the data [68], [75], [76]. The algebraic details will be published elsewhere. Finally, the distribution of Eq. 19 is recalculated while iteratively optimizing the global non-linear parameters of *f/f_0_* and 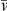, as well as the local non-linear parameters for the menisci *m_n_* (and bottom *b_n_* if the experiments contain back-diffusion signifying their dependence on *b_n_*).

Since the calculation of the sedimentation coefficient distribution is a constructive numerical approach, additional refinements can be made. For example, it is possible to use the global 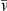 and the transformations Eqs. 16 and 17 only for species within a certain *s^(20,w)^* range, and use different (even empirical) 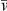-values and *_f/f0_*-values outside this range. This allows describing minor signal contributions from impurities and degradation products separately and eliminate their influence on the 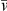 determination of the species of interest (as long as they sediment in a different range of sedimentation coefficients). These techniques were implemented in the software SEDPHAT, freely available from [47].

### Materials

Lyophylized bovine serum albumin (BSA) and phosphorylase B from rabbit muscle were purchased from Sigma-Aldrich (St. Louis, MO), and a buffered solution of ChromPur Human Immunoglobulin G (IgG) was purchased from Jackson ImmunoResearch (West Grove, PA). Water isotopically enriched to 97% H_2_^18^O was purchased from Cambridge Isotope Laboratories, Inc. (Andover, MA), and 99% enriched H_2_^18^O from Sigma-Aldrich. 10× stock solutions of phosphate buffered saline (PBS) was supplied by Cellgrow (Manassas, VA).

### Densimetry/Viscosimetry

Densimetry measurements were carried out in a DMA 5000 M density meter from Anton Paar (Graz, Austria) according to the method of Kratky et al. [26]. Viscosity measurements were performed using an AMVn automated micro-viscometer from Anton Paar.

Standard solutions of PBS were prepared by dilution of 10× PBS with either H_2_O, or isotopically-enriched H_2_^18^O, or both, delivered by micropipette, to have H_2_^18^0/H_2_O ratios of 0, 0.5, and 0.9 (v/v). The density and viscosity of the standards were measured at multiple temperatures between 4–20°C (Table 2).

**Table 2:** Partial-specific volumes obtained for different protein samples.

partial-specific volume (ml/g)	BSA	IgG	phosphorylase B
predicted/literature	0.733^a^	0.739^b^	0.737^a^
measured by densimetry^c^	0.730	0.764	0.742
global SV density variation: absorbance	n/d	0.734	0.743
average signal/noise ratio^d^		62 (0.32/0.005)	17 (0.065/0.004)
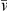 error estimate (+/−)^e^		0.004	0.014
global SV density variation: interference	0.732	0.728	0.734
average signal/noise ratio^d^	167 (0.6/0.004)	118 (0.6/0.005)	43 (0.12/0.0028)
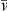 error estimate (+/−)^e^	0.001	0.001	0.005

a from amino acid composition predicted in SEDFIT;

b reported in [80];

c stock concentrations were determined spectrophotometrically using extinction coefficients as noted in Materials and Methods;

d average signal of sedimentation boundary *vs* average root-mean-square deviation of global fit;

e based on 68% confidence level.

For the measurement of the density increment 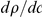, stock protein solutions at concentrations of between 9–20 mg/ml were dialyzed against PBS overnight at 4°C with 3 exchanges. Dilutions of the stock were prepared using the dialysate at a range of concentrations spanning 1 to 0.01 that of the stock. Final concentrations were determined spectrophotometrically at 280 nm, using extinction coefficients of 1.236 OD/(cm×mg/ml) for phosphorylase B (calculated from its amino acid composition), 0.6379 OD/(cm×mg/ml) for BSA determined by dry weight [16], and 1.36 OD/(cm×mg/ml) for IgG [77].

### Sedimentation Velocity

Sedimentation velocity experiments were performed according to standard procedures [78]. Sedimentation velocity samples were prepared by re-suspending the protein in 10× PBS buffer, and/or by dialysis against 10× PBS buffer. This stock was then diluted into H_2_O, or isotopically-enriched H_2_^18^O (based on 97% H_2_^18^O water for phorphorylase B and IgG, and on 99% H_2_^18^O water for BSA), such as to achieve H_2_^18^O/H_2_O ratios of 0, 0.5, and 0.9 (v/v). Sedimentation velocity samples (100 µl) were loaded into 3-mm double sector centerpieces with sapphire windows. (3 mm centerpieces were chosen in order to minimize the required sample volumes.) The reference sector was filled with equivalent buffer without heavy isotopes. Sedimentation was monitored at a rotor speed of 50,000 rpm and a temperature of 20°C using interference (IF) and/or absorbance optics at 280 nm. The sedimentation velocity profiles for proteins in solutions of increasing density were fit globally to obtain an estimate of the partial-specific volume using the “Hybrid Global Discrete Species Global Continuous Distribution” model in SEDPHAT (vs. 8.42), which allows for the global density variation analysis. For the analysis of absorbance data, only a single continuous segment was used for the analysis, whereas for interference data analysis a discrete species was added describing the signal from sedimentation of unmatched buffer salts. Error limits at a 68% confidence level were obtained using the surface projection method, i.e. by probing for the critical parameter values that, when fixed while all other unknowns are re-adjusted, cause an increase in the critical chi-square exceeding that tolerable on a given confidence level [79].

## Results

In order to test the performance of the global SV density variation method, we studied samples of three proteins – BSA, IgG, and phosphorylase B. The partial-specific volumes obtained by densimetry are listed in Table 2. For BSA and phosphorylase B the values agree well with the predictions from the amino acid composition.

For our IgG sample, the prediction was not easily possible due to the glycosylation of the protein, as well as the presence of different isotypes and different clones in the sample used. Further, the densimetric determination is similarly problematic due to the problem of extinction coefficients. For example, when using the extinction coefficient at 280 nm of 1.36 OD/(cm×mg/ml) [77], the measured density data result in a partial-specific volume of 0.764 ml/g. A higher extinction coefficient value of 1.40 OD/(cm×mg/ml) [80] would lead to a partial-specific volume of 0.757 ml/g, whereas lower values of 1.222 OD/(cm×mg/ml) [81] would result in a partial-specific volume of 0.788 ml/g. While this problem could be resolved by dry weight determination and the use of a monoclonal better defined sample, in the present context it highlights the strong dependency of the densimetric partial-specific volume on accurate extinction coefficient data, if the concentration is measured by absorption spectrophotometry.

The global SV density variation approach critically depends on accurate density and viscosity data for the different solvent conditions. Tabulated data for the density and viscosity of D_2_O and different H_2_O/D_2_O mixtures are available [82], [83]. Some experimental data is also published for H_2_^18^O [84]–[86], which has a viscosity at 20°C of 1.0641 cP, much closer to H_2_O than D_2_O (the latter having a value of 1.247 cP). Table 1 lists data experimentally determined for PBS in different dilutions of 97% isotope enriched H_2_^18^O, which we used for the sedimentation analysis.

We next conducted SV experiments with the protein samples diluted from stock into solutions of PBS with final water composition of 100% H_2_O, 50% and 10% (v/v) H_2_O/(97%)H_2_^18^O, to achieve solvent densities of 1.006 g/ml, 1.059 g/ml, and 1.103 g/ml, respectively. The SV experiments in different solvent densities were carried out side-by-side at 20°C and 50,000 rpm, scanned with either absorbance and/or interference optical system, and fit with a global model as described in Eqs. 13–19. Meniscus values, baseline and systematic noise contributions, as well as signal amplitude factors 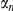 were adjustable local parameters, whereas the frictional ratio, the partial-specific volume, and the distribution *c*(*s*) were globally adjusted. In addition, for the IF data, buffer salt signals were modeled as discrete species with parameters globally refined. We observed error surfaces exhibiting local minima, and therefore alternated between Simplex and Marquardt-Levenberg optimization.

**Figure 1:**
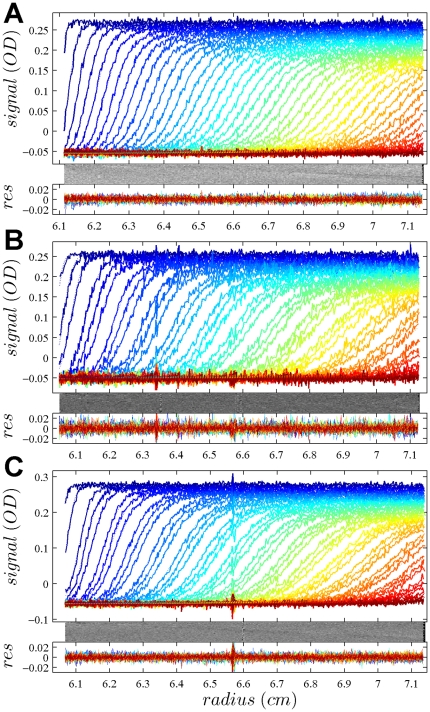
Global density variation SV analysis of the phosphorylase B sample recorded with the absorbance data at 280 nm. The sets of panels present the data in (A) H_2_O, (B) 50% H_2_^18^O, and (C) 90% H_2_^18^O based buffer. For each set of panels, the measured data (corrected for the time-invariant noise contributions) are shown as solid lines, and the global best-fit profiles are shown as thin dotted lines (virtually superimposed to the data). Higher color temperatures indicate later times. Below are the residuals bitmap (a 2d grey-scale representation of residual values with time plotted vertically and radius horizontally [69]) and the residuals of the fit, with rmsd of 0.00365 OD (A), 0.00411 OD (B), and 0.00380 (C). In the presence of H_2_^18^O, fewer scans were included into the analysis in order to achieve similar numbers of total scans representing the sedimentation process. The best-fit *c*(*s*) distribution from this analysis is shown in Figure 2.

**Figure 2:**
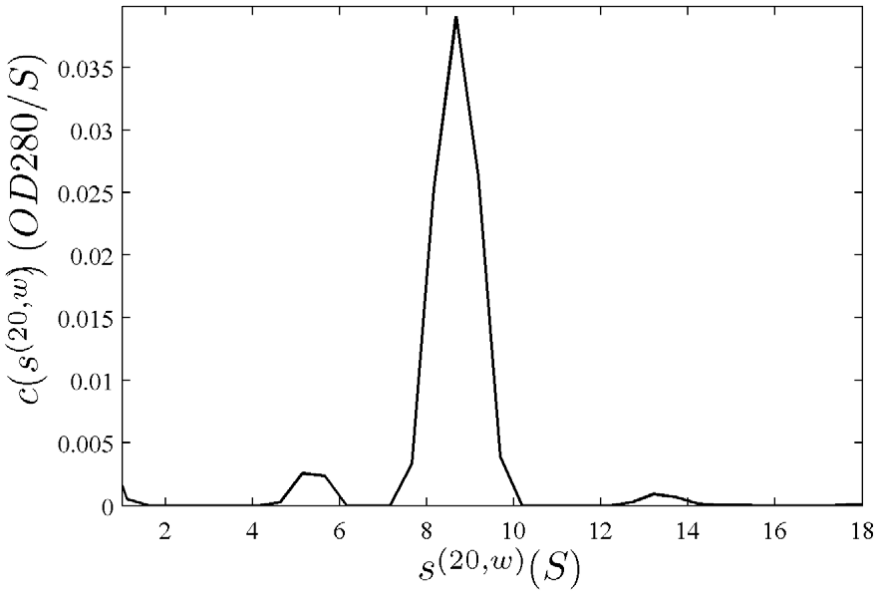
Sedimentation coefficient distribution for the global analysis of the absorbance data from the phosphorylase sample (**Figure 1**). Relative scaling factors 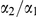 and 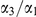 were 1.039 and 1.136 (see Eq. 19), indicating slightly different best-fit loading concentrations.

**Figure 3:**
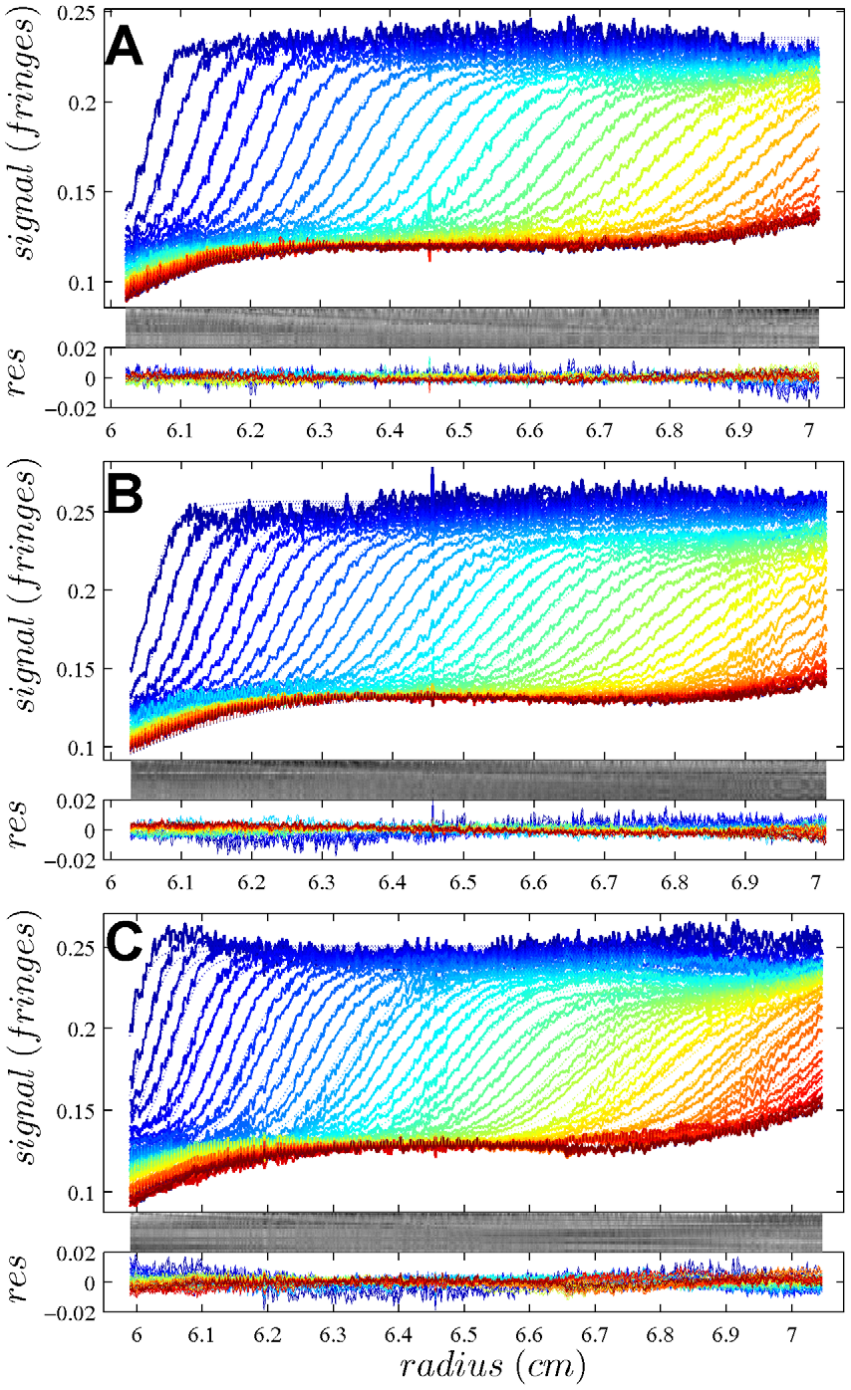
Interference optical data from the same sample as in **Figure 1**, in the same representation. Rmsd values are 0.00230 fringes (A), 0.00289 fringes (B) and 0.00312 fringes (C).

**Figure 4:**
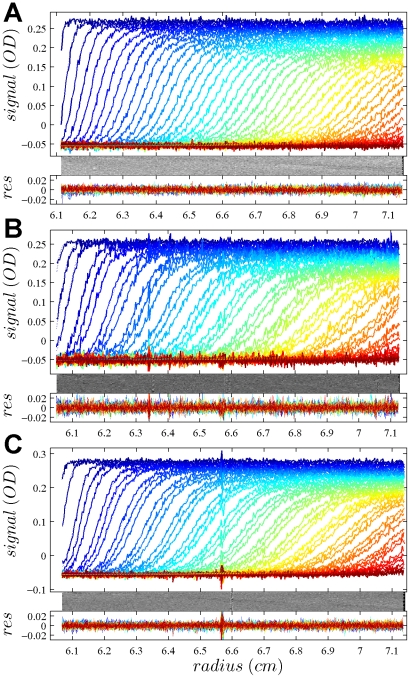
Global density variation SV analysis of the IgG sample recorded with the absorbance data at 280 nm. The sets of panels present the data in (A) H_2_O, (B) 50% H_2_^18^O, and (C) 90% H_2_^18^O based buffer. The presentation is analogous to that in Figure 1. Rmsd of the fit was 0.00462 OD (A), 0.00632 OD (B), and 0.00465 OD (C). The best-fit *c*(*s*) distribution from this analysis is shown in Figure 5, and the projections of the error surface in Figure 9.

**Figure 5:**
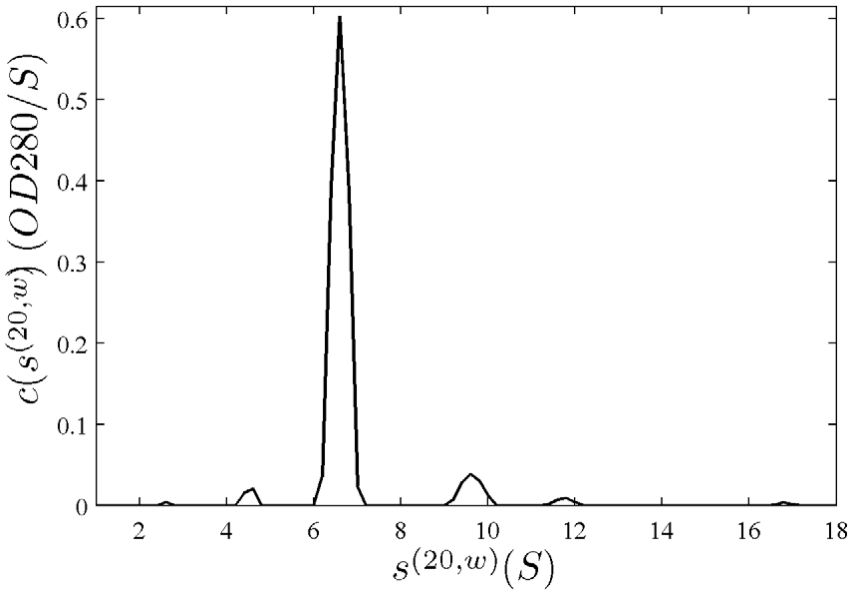
Sedimentation coefficient distribution for the global analysis of the absorbance data from the IgG sample (**Figure 4**). Relative scaling factors 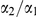 and 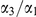 were 0.955 and 1.034, indicating slightly different best-fit loading concentrations.

**Figure 6:**
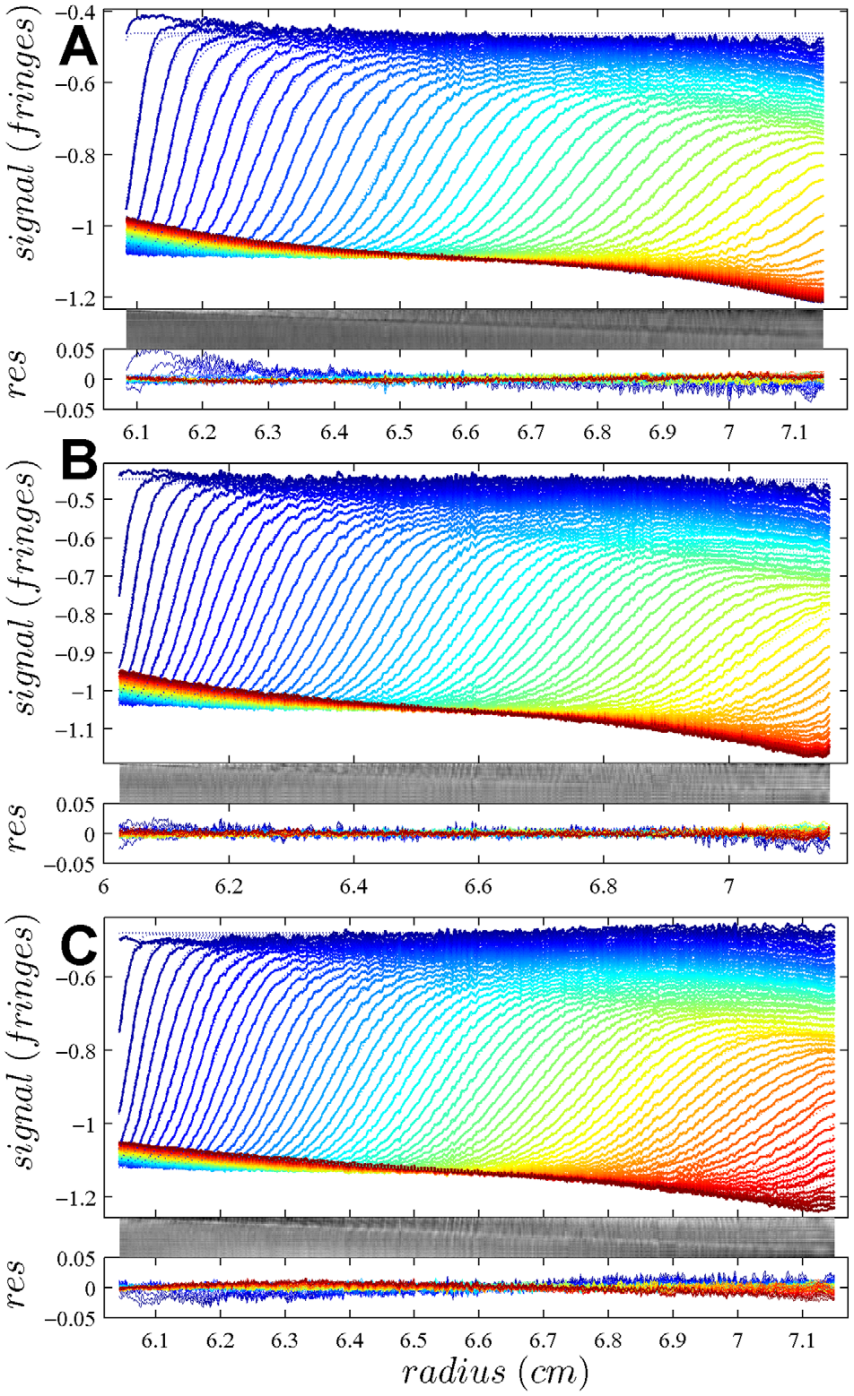
Interference optical data from the same sample as in **Figure 4**, in the same representation. Rmsd values are 0.00554 fringes (A), 0.00401 fringes (B) and 0.00575 fringes (C).

**Figure 7:**
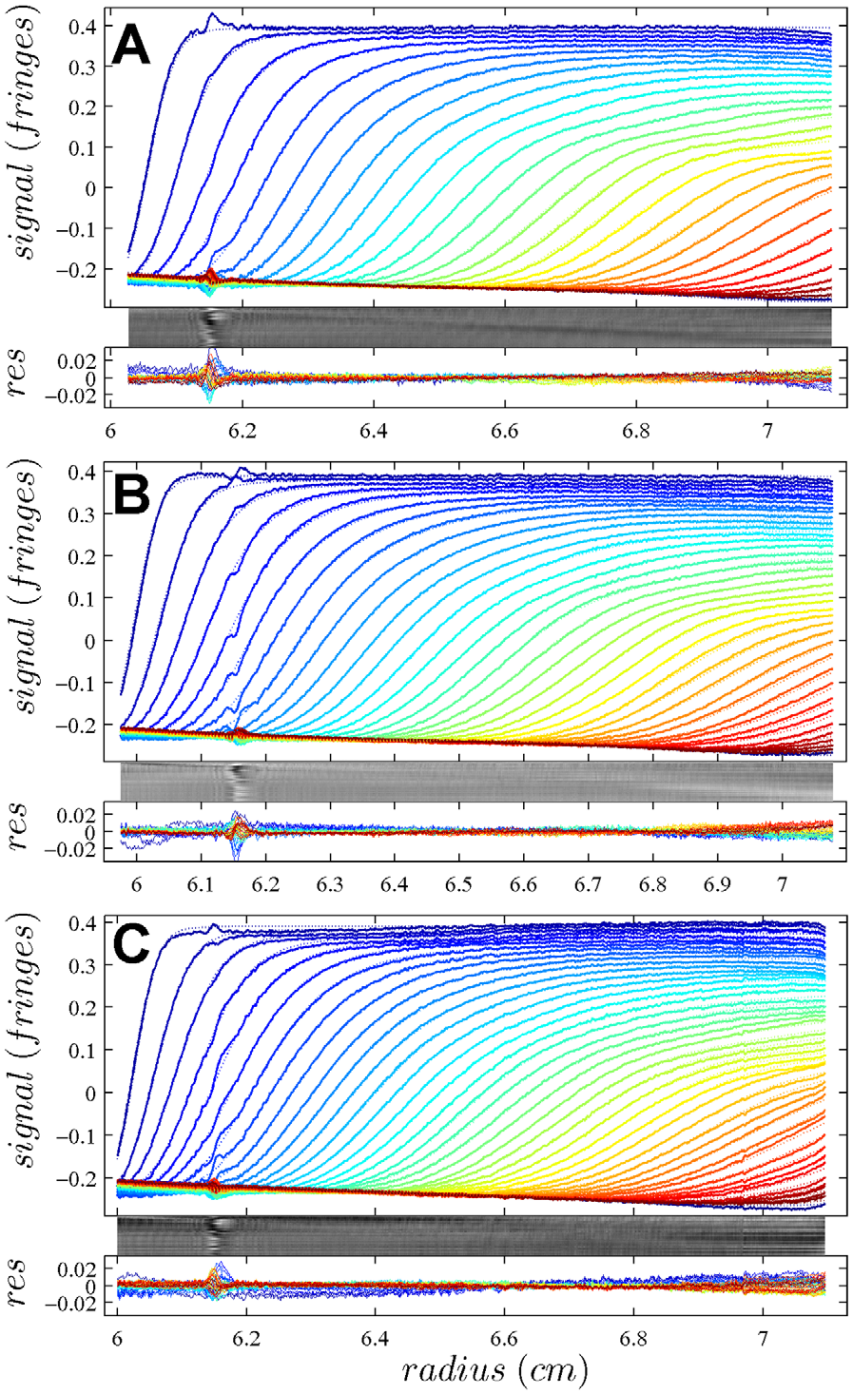
Global density variation SV analysis of the interference optical data from the BSA sample. The sets of panels present the data in (A) H_2_O, (B) 50% H_2_^18^O, and (C) 90% H_2_^18^O based buffer. The presentation is analogous to that in Figure 1. Rmsd of the fit was 0.00346 fringes (A), 0.00337 fringes (B), and 0.00416 fringes (C). The best-fit *c*(*s*) distribution from this analysis is shown in Figure 8, and the projections of the error surface in Figure 9.

**Figure 8:**
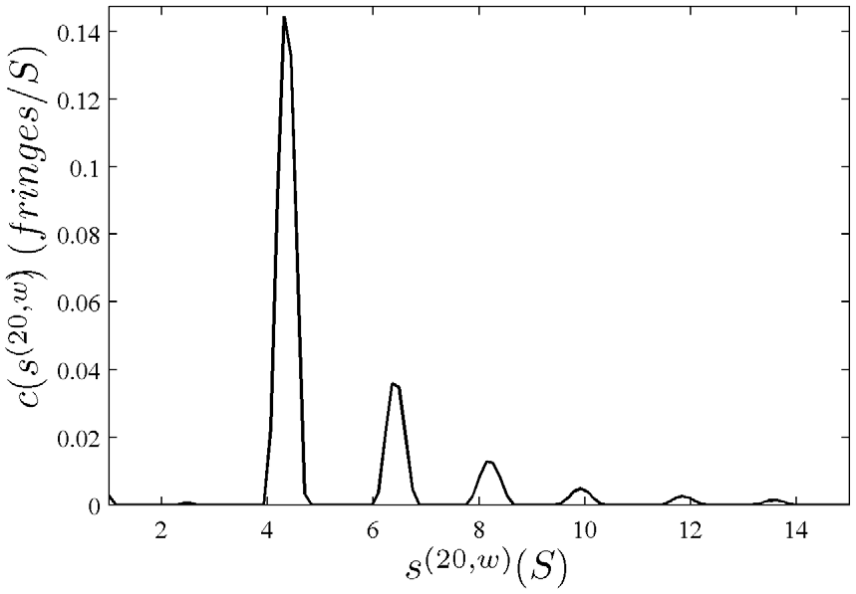
Sedimentation coefficient distribution for the global analysis of the BSA sample (**Figure 7**). Relative scaling factors 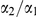 and 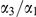 were 0.978 and 0.977, indicating slightly different best-fit loading concentrations.

In Figure 1, the raw data and the final quality of fit is shown for the absorbance data of the phosphorylase B sample. The corresponding c(s) distribution is shown in Figure 2, indicating some sample heterogeneity, and with the best-fit scaling factors reflecting a slight mismatch in the sample loading concentrations (probably due to pipetting errors). The interference data of the same sample is shown in Figure 3. It highlights the signal offsets arising from buffer mismatch, and the generally higher systematic component to the residuals arising from both remaining imperfections in the model and common instabilities in the data acquisition system. Nevertheless, in both data sets Figure 1 and Figure 3 the fit arrives at root-mean-square deviations (rmsd) that are very small and well within the usual noise of data acquisition. Similar is true for the fit to the absorbance (Figure 4 and Figure 5) and interference (Figure 6) IgG data, where the buffer mismatch led to negative signal offsets (which are not entirely captured in the model for the H_2_O data set). Likewise, the fit to the interference data of the BSA sample (Figure 7) shows common slight imperfections, small negative buffer offset captured in the model, but provides overall an excellent description of the boundaries with small rmsd close to the usual noise of data acquisition. The sedimentation coefficient distribution (Figure 8) exhibits a series of peaks from the well-known oligomers of BSA.

**Figure 9:**
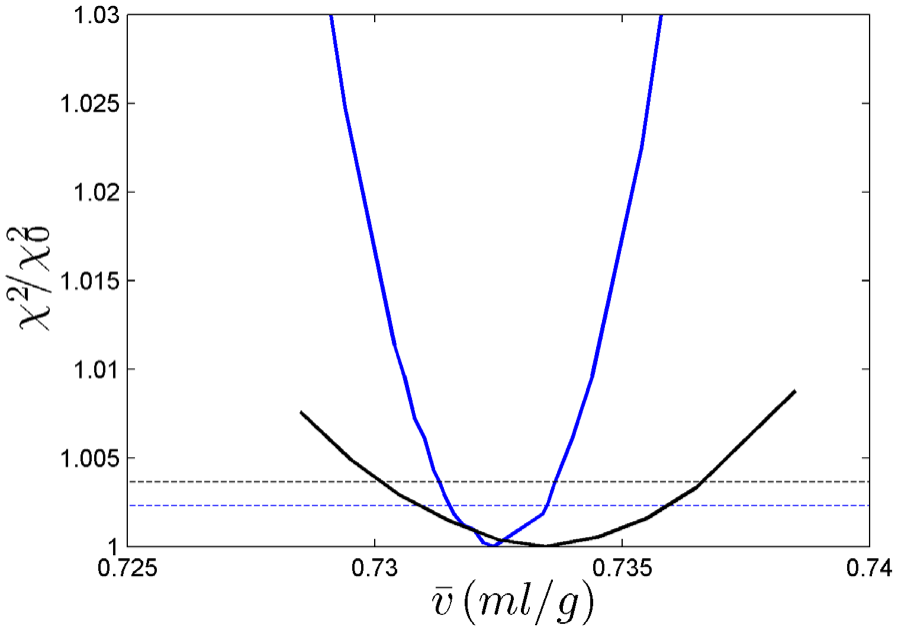
Projections of the error surface as a function of 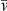
 **-values.** Shown are the relative increase in the χ^2^ of the fit as a function of different fixed 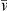-values, for each value freely adjusting all other unknown parameters [79]. Data are shown for the absorbance IgG data set (black) and the interference data set from the BSA sample (blue). For each, the dashed line shows the increase predicted by F-statistics for the 68% confidence level [79]. This critical increase of χ^2^ is lower for the BSA data set due to the significantly larger number of data points.

The best-fit 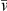-values from all density variation SV experiments are shown in Table 2. Generally, the values are in good agreement with those determined by composition or reported in the literature. Of particular interest are the statistical error estimates. Errors were estimated based on 68% confidence intervals and also reported in Table 2. Traces of the error surface projection as a function of 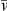 are shown in Figure 9. Not surprisingly, the errors are strongly dependent on the signal/noise ratio of the SV data. With signal/noise ratios >100, statistical errors were ∼0.001 ml/g, corresponding to relative errors of 0.14%. At signal/noise ratios of ∼50, relative errors of ∼0.6% were obtained. Generally, a signal/noise ratio of 100 can be achieved with loading signals of ∼0.5 fringes, corresponding for average proteins in 12 mm centerpieces to concentrations of 0.15 mg/ml, or 0.6 mg/ml in 3 mm centerpieces. In order to generate the 10–12 mm solution columns we used, for either centerpiece approximately ∼60 µg of protein is required.

We analyzed the absorbance and interference data independently, in order to judge consistency (Table 2). While for the phosphorylase B the error intervals from absorbance and interference analysis overlap, they are just non-overlapping for the IgG data. Although there could be systematic deviations causing this slight discrepancy, the data do not show a clear indication of this, as can be discerned from the residuals overlay and bitmap. A global analysis of the data from both optical systems is possible. For phosphorylase B, for example, this results in a best-fit 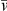-value of 0.735 ml/g. This is close to the value of the interference data due to its better signal/noise ratio and the higher number of data points. In principle, correction factors could be applied in SEDPHAT to modify the relative weights.

## Discussion

Many biophysical solution methods require knowledge about the partial-specific volume of the macromolecule studied. The present work is focused mainly on AUC, a technique that offers several possibilities to determine this parameter. Recent work by Gohon *et al.* [33] described the use of SV density variation in conjunction with relatively high resolution sedimentation coefficient distributions obtained by direct boundary modeling, and demonstrated the advantages of this approach. Nury *et al.* [53] exploited this approach to determine unambiguously the association state of a solubilized membrane protein. In the current work, we aimed at developing a convenient analysis platform for this technique, and increasing the precision of the analysis by exploiting direct fitting and global analysis principles. This allows deriving information of changes in the macromolecular buoyancy from both *s-*values and buoyant molar masses simultaneously. From the initial applications to test systems, it appears the precision of the determination of 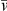-values for proteins can be very high. When SV data had a high signal/noise ratio, we obtained statistical error estimates of as low as ±0.001 ml/g that compare favorably to densimetry and SE density variation. This statistical error of 0.14% is consistent with the statistical precision of relative *s-*values of up to 0.1% [67].

The density variation SV approach has specific advantages in comparison with densimetry, which has some limitations in applicability, chiefly due to the relatively large sample volumes required [29], [40], [43], and in some cases due to practical requirements of sample preparation [33] and the required knowledge of protein weight concentrations. In principle, it can be very accurate and provide 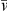-values for proteins to within 0.001 ml/g with 6-decimal place instruments [26]. However, much larger errors can arise when accounting for possible systematic errors in the protein weight concentrations used [28], which could commonly arise from uncertainties in the extinction coefficients: assuming an error in concentration (or extinction coefficients) of 2%, the resulting error in 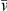 grows to 0.005 ml/g [26]. The effect of the uncertainty in the exact protein concentration is very significant when the latter is based on extinction coefficients predicted from the amino acid sequence, which were estimated to be in error, on average, by ∼4% [87]. (On the other hand, this sensitivity means that if the partial-specific volume is known accurately from other methods, then densimetry could be used effectively to determine protein concentrations and extinction coefficients.) Even though the dry-weight approach could be exploited to determine the protein concentration more accurately, the requirement for a large quantity of soluble material and the non-trivial measurement process make this approach impractical for many or most proteins.

The density variation technique for SE described by Edelstein & Schachman [25] addresses some of the major limitations, both in requirements for sample amounts and their exact concentration. The errors in protein 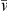-values were estimated to be 0.003 ml/g under ideal circumstances [25]. A major drawback is that high sample purity and stability is essential [33]. This is because for heterogeneous mixtures, the measured ‘average’ molar mass is an ill-defined average that, due to different radial distribution of high- and low-molar mass species in conditions of different buoyancy, will likely significantly depend on solvent density, thereby skewing the density variation analysis. This renders this approach not applicable for many polymer and nano-particle systems.

As shown by Gohon, it is one of the strengths of SV that it is applicable to imperfectly or even poorly purified, heterogeneous samples [24], [33]. While requiring similar sample amounts as SE, and no knowledge of the exact protein concentration, it should work well as long as a sedimentation boundary can be attributed to the macromolecule of interest. (For example, taking advantage of the multi-segmented design of the sedimentation coefficient distributions that can attribute different 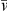-values to species with different sedimentation coefficient.) Further, for relatively pure material, intrinsically the measurement of differences in *s-*values (0.1% [67]) is far more precise than the buoyant molar mass from analysis of sedimentation equilibrium.

Since it is not necessary to resolve individual peaks in the global distribution fit approach, this opens the density variation to the study of macromolecules with intrinsically broad size-distributions. Furthermore, it should also be applicable to reversibly interacting systems. The diffusion deconvoluted sedimentation coefficient distributions lend themselves very well to fit the sedimentation boundaries of reversibly interacting systems, if properly interpreted. This has been shown by numerous applications, and was recently theoretically explained in the intuitive framework of effective particle theory [7], [88]. To the extent that the SV experiment can be carried out using the same loading concentration at different densities, and using a density contrast method that does not affect homo- or hetero-associations (which we expect, e.g., from H_2_^18^O but not obviously in D_2_O), the sedimentation coefficient distribution will be invariant and the determination of an average 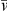-value from a global fit should be possible. Due to the relative insensitivity of mass action law to the total concentration, even small differences in loading concentration may not propagate much into differences in 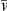-value.

(To estimate the required precision in loading concentration for associating systems, let us consider at 50 kDa self-associating protein with partial-specific volume of 0.73 ml/g, monomer s-value of 3.5 S, and dimer *s*-value of 5.5 S. At loading concentration at 3*K_D_* (where monomer and dimer are equally populated on a molar basis and the isotherm is steepest), we would observe weighted average *s_w_*-values of 4.833 S in H_2_O and a 3.177 S in H_2_^18^O. If the latter measurement was done (unknowingly) at a 5% higher total protein concentration, the dimer fraction would increase by (0.96%, and a *s_w_*-value of 3.185 S would be measured. If that error was not caught, it would lead to 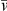-value of 0.7288 ml/g rather than the correct value of 0.7300 ml/g, i.e. propagate to only a 0.2% error in 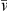. In comparison, the same system at the same concentration in SE at 15,000 rpm, assuming a 4 mm solution column from 6.8 cm to 7.2 cm that can be evaluated from 6.81 cm to 7.13 cm, the apparent ‘cell-average’ buoyant molar mass in H_2_O is 20,581 Da, and 15,574 Da in H_2_^18^O. Even without concentration error, the corresponding ‘cell-average’ molar mass values at correct 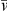 are different with 75.9 kDa and 82.3 kDa, respectively, due to the different radial localization within and outside the analysis range of the monomer and dimer. If the self-association is unrecognized and the density variation analysis is applied, the resulting apparent 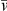 would be only 0.685 mg/ml – qualitatively wrong. This is only slightly exacerbated with a 5% higher concentration in the H_2_^18^O *vs* the H_2_O experiment, which would lead to a 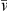-value of 0.681 mg/ml.)

In practice, there are several options to achieve a density variation. Most importantly, they should be chosen such that the partial-specific volume of the sedimenting particle remains unchanged, i.e. considering an invariant sedimenting particle. (This obviously excludes, for example, combinations into one global density contrast experiment of densifying co-solute not penetrating the hydration shell, and solvent isotope approaches that do partition evenly into the hydration layer.) Further, dependent on the strategy, different partial-specific volumes are determined and different sedimenting particles are implied. When using densifying co-solvents, those that have been found experimentally to leave hydrated proteins as invariant particles, defined with a constant partial-specific volume and water binding in a large range of co-solvent concentrations, seem best suitable (e.g., sucrose), whereas others show more complex behavior, including glycerol and trehalose, and therefore are of more uncertain use for the purpose of density variation SV [36].

When using heavy water containing deuterium, H-D exchange must be taken into consideration, otherwise errors for proteins estimated to 4–6% will be incurred [24]. As an alternative, Rowe and colleagues have recently demonstrated the use of H_2_^18^O [51], which has very similar density as D_2_O but not as large a relative viscosity. This eliminates the need for H-D exchange corrections, as well as potential effects from different hydrogen bond strengths and potential effects on protein interactions [89]. Unfortunately, the currently ∼100-fold higher cost of H_2_^18^O over D_2_O makes it impractical to use with dialysis equilibrium. This problem may be mitigated to some extent by dialysis of a sample stock against H_2_O-based buffer and dilution into the H_2_^18^O-based solvent of the same composition. This is not completely rigorous, and may cause errors with macromolecules that weakly bind buffer components if the composition of the buffer changes upon dilution of the protein. (On the other hand, strong interactions might remain saturated after dilution [45].) While we have used H_2_^18^O in the current work with good results for the small set of test proteins, H-D corrections for working with D_2_O have also been implemented in SEDPHAT [47], which should allow to circumvent this possible problem.

A clear drawback of density variation SV is the dependence on accurate buffer viscosity data. For a given buffer composition and temperature, the viscosity measurement needs to be carried out very carefully only once and then be tabulated for future reference. Further, in the present approach, this is slightly alleviated by the fact that, simultaneous to the sedimentation coefficient, also the boundary spread is modeled in terms of a density-adjusted buoyant molar mass, the latter being dependent on the ratio 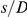 which is independent of sample viscosity.

The dual source of information from boundary spread and boundary migration allows for different strategies to experimentally emphasize either diffusion at lower rotor speeds or sedimentation at higher rotor speeds. We have not compared which approach is better, but since the resolution improves with higher rotor speed, high rotor speeds are likely most advantageous in most cases. However, different rotor speeds can be naturally included and globally fit in our implementation in SEDPHAT. In that case, to ensure consistency in temperature calibration and radial calibration, such data should be collected with the same instrument and without radial calibration in between. Similarly, a global analysis of data from different optical systems is possible, in principle, although we believe that no significant further gain in accuracy may result, due to the dissimilar number of data points, their different noise structure and susceptibility to systematic errors, and possible slight inconsistencies in radial calibration to which the present analysis would be particularly sensitive.

## References

[1] Eisenberg H (1981). Forward scattering of light, X-rays and neutrons.. Q Rev Biophys.

[2] Zaccai G, Jacrot B (1983). Small angle neutron scattering.. Annu Rev Biophys Bioeng.

[3] Ebel C, Schuck P (2007). Solvent mdeidated protein-protein interactions.. Protein Interactions: Biophysical Approaches for the study of complex reversible systems.

[4] Svedberg T, Pedersen KO (1940). The ultracentrifuge.

[5] Correia JJ (2000). Analysis of weight average sedimentation velocity data.. Methods in Enzymology.

[6] Schuck P (2003). On the analysis of protein self-association by sedimentation velocity analytical ultracentrifugation.. Anal Biochem.

[7] Schuck P (2010). Sedimentation patterns of rapidly reversible protein interactions.. Biophys J.

[8] Zhao H, Balbo A, Brown PH, Schuck P (2011). The boundary structure in the analysis of reversibly interacting systems by sedimentation velocity.. Methods.

[9] Veronese FM (2001). Peptide and protein PEGylation: a review of problems and solutions.. Biomaterials.

[10] Heredia KL, Maynard HD (2007). Synthesis of protein-polymer conjugates.. Organic & Biomolecular Chemistry.

[11] Arnold MS, Suntivich J, Stupp SI, Hersam MC (2008). Hydrodynamic characterization of surfactant encapsulated carbon nanotubes using an analytical ultracentrifuge.. ACS Nano.

[12] Shu JY, Tan C, DeGrado WF, Xu T (2008). New design of helix bundle peptide-polymer conjugates.. Biomacromolecules.

[13] Wandrey C, Hasegawa U, van der Vlies AJ, O'Neil C, Angelova N (2010). Analytical ultracentrifugation to support the development of biomaterials and biomedical devices.. Methods.

[14] Aragon S (2011). Recent advances in macromolecular hydrodynamic modeling.. Methods.

[15] Howlett GJ, Minton AP, Rivas G (2006). Analytical ultracentrifugation for the study of protein association and assembly.. Curr Opin Chem Biol.

[16] Zhao H, Brown PH, Schuck P (2011). On the distribution of protein refractive index increments.. Biophys J.

[17] Schachman HK, Lauffer MA (1949). The hydration, size and shape of tobacco mosaic virus.. J Am Chem Soc.

[18] Casassa EF, Eisenberg H (1964). Thermodynamic analysis of multicomponent systems.. Adv Protein Chem.

[19] Inoue H, Timasheff SN (1968). The interaction of beta-lactoglobulin with solvent components in mixed water-organic solvent systems.. J Am Chem Soc.

[20] Cohn EJ, Edsall JT, Cohn EJ, Edsall JT (1943). Density and apparent specific volume of proteins.. Proteins, Amino Acids and Peptides.

[21] McMeekin TL, Marshall K (1952). Specific volumes of proteins and the relationship to their amino acid contents.. Science.

[22] Cheng PY, Schachman HK (1955). Studies on the validity of the Einstein viscosity law and Stokes' law of sedimentation.. Journal of Polymer Science.

[23] Martin WG, Cook WH, Winkler CA (1956). The determination of partial specific volumes by differential sedimentation.. Canadian Journal of Chemistry.

[24] Martin WG, Winkler CA, Cook WH (1959). Partial specific volume measurements by differential sedimentation.. Canadian Journal of Chemistry.

[25] Edelstein J, Schachman H (1967). Simultaneous determination of partial specific volumes and molecular weights with microgram quantities.. J Biol Chem.

[26] Kratky O, Leopold H, Stabinger H (1973). The determination of the partial-specific volume of proteins by the mechanical oscillator technique.. Methods in Enzymology.

[27] Hunter MJ (1978). Partial specific volume measurements using the glass diver.. Methods Enzymol.

[28] Stothart PH (1984). Determination of partial specific volume and absolute concentration by densimetry.. Biochem J.

[29] Durchschlag H, Hinz H-J (1986). Specific volumes of biological macromolecules and some other molecules of biological interest.. Thermodynamic data for biochemistry and biotechnology.

[30] Perkins SJ (1986). Protein volumes and hydration effects. The calculations of partial specific volumes, neutron scattering matchpoints and 280-nm absorption coefficients for proteins and glycoproteins from amino acid sequences.. Eur J Biochem.

[31] Schilling K, Coelfen H (1999). Application of the solvent density variation method to sedimentation velocity experiments on biological systems.. ProgColloid Polymer Sci.

[32] Schuck P (2004). A model for sedimentation in inhomogeneous media. I. Dynamic density gradients from sedimenting co-solutes.. Biophys Chem.

[33] Gohon Y, Pavlov G, Timmins P, Tribet C, Popot JL (2004). Partial specific volume and solvent interactions of amphipol A8–35.. Anal Biochem.

[34] Eisenberg H (1992). Halophilic malate dehydrogenase - a case history of biophysical investigations: ultracentrifugation, light-, x-ray and neutron scattering.. Biochem Soc Symp.

[35] Reynolds JA, Tanford C (1976). Determination of molecular weight of protein moiety in protein-detergent complexes without prior knowledge of detergent binding.. Proc Nat Acad Sci.

[36] Ebel C, Eisenberg H, Ghirlando R (2000). Probing protein-sugar interactions.. Biophys J.

[37] Arakawa T (1986). Calculation of the partial specific volumes of proteins in concentrated salt, sugar, and amino acid solutions.. J Biochem.

[38] Gekko K, Timasheff SN (1981). Mechanism of protein stabilization by glycerol: preferential hydration in glycerol-water mixtures.. Biochemistry.

[39] Elder JP (1979). Density measurements by the mechanical oscillator.. Methods Enzymol.

[40] Lee JC, Gekko K, Timasheff SN (1979). Measurements of preferential solvent interactions by densimetric techniques.. Methods Enzymol.

[41] Eisenberg H (2000). Analytical ultracentrifugation in a Gibbsian perspective.. Biophys Chem.

[42] Eisenberg H (2003). Modern analytical ultracentrifugation in protein science: look forward, not back.. Protein Sci.

[43] Lebowitz J, Lewis MS, Schuck P (2003). Back to the future: A rebuttal to Henryk Eisenberg.. Protein Sci.

[44] Tziatzios C, Schuck P, Schubert D, Tsiotis G (1994). The molar mass of an active photosystem-I complex from the cyanobacterium synechococcus PCC-7002.. Zeitschrift f Naturforschung C.

[45] Hellman LM, Rodgers DW, Fried MG (2010). Phenomenological partial-specific volumes for G-quadruplex DNAs.. Eur Biophys J.

[46] Noy D, Calhoun JR, Lear JD (2003). Direct analysis of protein sedimentation equilibrium in detergent solutions without density matching.. Anal Biochem.

[47] Schuck P (2011). https://sedfitsedphat.nibib.nih.gov/software/default.aspx.

[48] Lustig A, Engel A, Zulauf M (1991). Density determination by analytical ultracentrifugation in a rapid dynamic gradient - application to lipid and detergent aggregates containing proteins.. Biochem Biophys Acta.

[49] Mayer G, Ludwig B, Muller HW, van den Broek JA, Friesen RHE (1999). Studying membrane proteins in detergent solution by analytical ultracentrifugation:different methods for density matching.. ProgColloid Polymer Sci.

[50] Center RJ, Schuck P, Leapman RD, Arthur LO, Earl PL (2001). Oligomeric Structure of Virion-Associated and Soluble Forms of the Simian Immunodeficiency Virus Envelope Protein in the Pre-Fusion Activated Conformation.. Proc Natl Acad Sci U S A.

[51] Mullin NP, Yates A, Rowe AJ, Nijmeijer B, Colby D (2008). The pluripotency rheostat Nanog functions as a dimer.. Biochem J.

[52] Gagen WL (1966). The significance of the “partial specific volume” obtained from sedimentation data.. Biochemistry.

[53] Nury H, Manon F, Arnou B, le Maire M, Pebay-Peyroula E (2008). Mitochondrial bovine ADP/ATP carrier in detergent is predominantly monomeric but also forms multimeric species.. Biochemistry.

[54] Gohon Y, Dahmane T, Ruigrok RW, Schuck P, Charvolin D (2008). Bacteriorhodopsin/amphipol complexes: structural and functional properties.. Biophys J.

[55] Eisenberg H (1976). Biological macromolecules and polyelectrolytes in solution.

[56] Ebel C, Costenaro L, Pascu M, Faou P, Kernel B (2002). Sovent interactions of halophilic malate dehydrogenase.. Biochemistry.

[57] Harpaz Y, Gerstein M, Chothia C (1994). Volume changes on protein folding.. Structure.

[58] Svedberg T, Pedersen KO (1940). Die Ultrazentrifuge.

[59] Tardieu A, Vachette P, Gulik A, le Maire M (1981). Biological macromolecules in solvents of variable density: characterization by sedimentation equilibrium, densimetry, and X-ray forward scattering and an application to the 50S ribosomal subunit from Escherichia coli.. Biochemistry.

[60] Salvay AG, Santamaria M, LeMaire M, Ebel C (2007). Analytical ultracentrifugation sedimentation velocity for the characterization of detergent-solubilized membrane proteins Ca++-ATPase and ExbB.. J Biol Phys.

[61] le Maire M, Arnou B, Olesen C, Georgin D, Ebel C (2008). Gel chromatography and analytical ultracentrifugation to determine the extent of detergent binding and aggregation, and Stokes radius of membrane proteins using sarcoplasmic reticulum Ca2+-ATPase as an example.. Nat Protoc.

[62] Ebel C (2011). Sedimentation velocity to characterize surfactants and solubilized membrane proteins.. Methods.

[63] Schubert D, Schuck P (1991). Analytical ultracentrifugation as a tool for studying membrane proteins.. Progr Colloid Polym Sci.

[64] Howlett GJ, Harding SE, Rowe AJ, Horton JC (1992). Sedimentation analysis of membrane proteins.. Analytical Ultracentrifugation in Biochemistry and Polymer Science.

[65] Eisenberg H, Felsenfeld G (1981). Hydrodynamic studies of the interaction between nucleosome core particles and core histones.. J Mol Biol.

[66] Eisenberg H, Saenger W (1990). Solution properties of DNA: sedimentation, scattering of light, x-rays and neutrons, and viscometry.. Numerical data and functional relationships in science and technology.

[67] Errington N, Rowe AJ (2003). Probing conformation and conformational change in proteins is optimally undertaken in relative mode.. Eur Biophys J.

[68] Schuck P (2000). Size distribution analysis of macromolecules by sedimentation velocity ultracentrifugation and Lamm equation modeling.. Biophys J.

[69] Dam J, Schuck P (2004). Calculating sedimentation coefficient distributions by direct modeling of sedimentation velocity profiles.. Methods Enzymol.

[70] Schuck P (2009). On computational approaches for size-and-shape distributions from sedimentation velocity analytical ultracentrifugation.. Eur Biophys J.

[71] Lamm O (1929). Die Differentialgleichung der Ultrazentrifugierung.. Ark Mat Astr Fys.

[72] Brown PH, Schuck P (2008). A new adaptive grid-size algorithm for the simulation of sedimentation velocity profiles in analytical ultracentrifugation.. Comput Phys Commun.

[73] Schuck P, Demeler B (1999). Direct sedimentation analysis of interference optical data in analytical ultracentrifugation.. Biophys J.

[74] Schuck P (2010). Some statistical properties of differencing schemes for baseline correction of sedimentation velocity data.. Anal Biochem.

[75] Brown PH, Balbo A, Schuck P (2009). On the analysis of sedimentation velocity in the study of protein complexes.. Eur Biophys J.

[76] Phillips DL (1962). A technique for the numerical solution of certain integral equations of the first kind.. Assoc Comput Mach.

[77] Page M, Thorpe R, Walker JM (2002). Purification of IgG using DEAE-sepharose chromatography.. The Protein Protocols Handbook. 2nd Edition ed.

[78] Brown PH, Balbo A, Schuck P (2008). Characterizing protein-protein interactions by sedimentation velocity analytical ultracentrifugation.. Curr Protoc Immunol.

[79] Bevington PR, Robinson DK (1992). Data Reduction and Error Analysis for the Physical Sciences.

[80] Jossang T, Feder J, Rosenqvist E (1988). Photon correlation spectroscopy of human IgG.. J Protein Chem.

[81] Tewari UJ, Mukkur TK (1975). Isolation and physico-chemical characterization of bovine serum and colostral immunoglobulin G (IgG) subclasses.. Immunochemistry.

[82] Jones G, Fornwalt HJ (1936). The viscosity of deuterium oxide and its mixtures with water at 25C.. J Chem Phys.

[83] Matsunaga N, Nagashima A (1983). Transport properties of liquid and gasous D2O over a wide range of temperature and pressure.. J Phys Chem Ref Data.

[84] Steckel F, Szapiro S (1963). Physical properties of heavy oxygen water. Part 1. - Density and thermal expansion.. Trans Faraday Soc.

[85] Kudish AI, Wolf D (1972). Physical properties of heavy-oxygen water.. J Am Chem Soc - Faraday Trans I.

[86] Kudish AI, Wolf D, Steckel F (1973). Correction.. JCS Faraday I.

[87] Pace CN, Vajdos F, Fee L, Grimsley G, Gray T (1995). How to measure and predict the molar absorption coefficient of a protein.. Protein Sci.

[88] Schuck P (2010). Diffusion of the reaction boundary of rapidly interacting macromolecules in sedimentation velocity.. Biophys J.

[89] Woodfin BM, Henderson RF, Henderson TR (1970). Effects of D2O on the association-dissociation equilibrium in subunit proteins.. J Biol Chem.

